# Is the HIV burden in India being overestimated?

**DOI:** 10.1186/1471-2458-6-308

**Published:** 2006-12-20

**Authors:** Lalit Dandona, Vemu Lakshmi, G Anil Kumar, Rakhi Dandona

**Affiliations:** 1 Health Studies Area, Centre for Human Development, Administrative Staff College of India, Hyderabad, India; 2 Department of Microbiology, Nizam's Institute of Medical Sciences, Hyderabad, India

## Abstract

**Background:**

The HIV burden estimate for India has a very wide plausibility range. A recent population-based study in a south Indian district demonstrated that the official method used in India to estimate HIV burden in the population, which directly extrapolates annual sentinel surveillance data from large public sector antenatal and sexually transmitted infection (STI) clinics, led to a 2–3 times higher estimate than that based on population-based data.

**Methods:**

We assessed the generalisability of the reasons found in the Guntur study for overestimation of HIV by the official sentinel surveillance based method: addition of substantial unnecessary HIV estimates from STI clinics, the common practice of referral of HIV positive/suspect patients by private practitioners to public hospitals, and a preferential use of public hospitals by lower socioeconomic strata. We derived conservative correction factors for the sentinel surveillance data and titrated these to the four major HIV states in India (Andhra Pradesh, Maharashtra, Karnataka and Tamil Nadu), and examined the impact on the overall HIV estimate for India.

**Results:**

HIV data from STI clinics are not used elsewhere in the world as a component of HIV burden estimation in generalised epidemics, and the Guntur study verified that this was unnecessary. The referral of HIV positive/suspect patients from the private to the public sector is a widespread phenomenon in India, which is likely causing an upward distortion in HIV estimates from sentinel surveillance in other parts of India as well. Analysis of data from the nationwide Reproductive and Child Health Survey revealed that lower socioeconomic strata were over-represented among women seeking antenatal care at public hospitals in all major south Indian states, similar to the trend seen in the Guntur study. Application of conservative correction factors derived from the Guntur study reduced the 2005 official sentinel surveillance based HIV estimate of 3.7 million 15–49 years old persons in the four major states to 1.5–2.0 million, which would drop the official total estimate of 5.2 million 15–49 years old persons with HIV in India to 3–3.5 million.

**Conclusion:**

Plausible and cautious extrapolation of the trends seen in a recent large and rigorous population-based study of HIV in a south Indian district suggests that India is likely grossly overestimating its HIV burden with the current official sentinel surveillance based method. This method needs revision.

## Background

UNAIDS recently estimated that in 2005 India had 5.7 million persons of all ages living with HIV, the highest number for any country in the world, but because of poor confidence in the HIV estimation data from India the plausibility range of this estimate is almost three-fold from 3.4 to 9.4 million [1]. The official figure for the number of persons with HIV in India estimated by its National AIDS Control Organization (NACO), based on sentinel surveillance data of 2005 from public hospitals, is 5.2 million [2]. The modest difference between the UNAIDS and NACO estimates is likely due to the former including all age groups and the latter estimating HIV up to 49 years of age. The important issue however is that reliable data from population-based studies of HIV would help improve the HIV estimates for India, which has been acknowledged even officially [3,4].

We have recently reported data from a large-sample methodologically rigorous population-based study of HIV in the relatively high prevalence Guntur district of the south Indian state of Andhra Pradesh [5]. A systematic comparison of the HIV estimate from this study and that based on the official sentinel surveillance method revealed that the latter overestimated the HIV burden in this district 2–3 times as compared with the population-based data adjusted for under-represented high-risk groups. The reasons for this overestimation in the order of importance were: addition of substantial unnecessary HIV estimates from STI clinics in the sentinel surveillance method, the common practice of referral of HIV positive and suspect patients by private practitioners to public hospitals including antenatal clinics, and a preferential use of public hospitals by lower socioeconomic strata that had higher HIV prevalence in this study. In this paper, we use cautious assumptions derived from the comparison in the Guntur study, and use the latest available nationwide data on the patterns of antenatal care utilisation, to attempt an indicative plausible range of HIV estimates in the four major Indian states that are estimated to contribute the bulk of HIV burden in India.

## Methods

We assessed, using the best available information, the degree to which the reasons for overestimation of HIV burden by the official sentinel surveillance method found in the Guntur study could be extrapolated to the four Indian states of Andhra Pradesh, Karnataka, Maharashtra and Tamil Nadu, which together contributed 72% of the HIV burden in India according to the sentinel surveillance of 2005 [2].

We studied information about methods of HIV estimation in the population globally and in India [1,2,4,6,7]. We assessed if HIV data from STI clinics were used for HIV estimation in generalised epidemics elsewhere. We probed the issue of referral of HIV positive or suspect persons by private practitioners to public hospitals in India. We analysed data from the nationwide Reproductive and Child Health District Level Household Survey of 2002–2004 [8] to understand the antenatal care utilisation trends, and the standard of living index (SLI) distribution of women using public hospital antenatal care, in all states of India classified by NACO as high HIV prevalence states and all other states with an estimated population of more than 25 million in the year 2005.

We used conservative extrapolations from the Guntur study data regarding the distortion caused in HIV prevalence among women utilising antenatal care at public hospitals in the four major HIV states in India due to referral of HIV positive and suspect women to public hospitals and due to the SLI distribution of women using pubic hospital antenatal care. Although in the Guntur study we found a doubling of the HIV prevalence in women using public hospital antenatal clinics due to referrals [5], we assumed conservatively that this upward distortion would range from 1.25 to 1.5 times in the four major HIV states in India. While in the Guntur study we found that the HIV rate was 2.3 times in women belonging to the lower half of SLI as compared with those belonging to the upper half [5], we assumed that the Guntur estimate may be the upper limit and that this ratio may range between 1.5 and 2.3. We used these assumptions to adjust the antenatal HIV prevalence from the 2005 sentinel surveillance in the four major HIV states in India [2], for possible distortions due to HIV referrals to public hospitals and HIV differential by SLI, taking into account the SLI distribution of public hospital antenatal care users based on the Reproductive and Child Health District Level Household Survey data.

To arrive at population estimates of HIV burden we also upward adjusted the antenatal HIV prevalence for high-risk groups that may be under-represented among antenatal women. In the Guntur study, inclusion of under-represented high-risk groups increased the population-based HIV prevalence in adults from 1.72% to 1.79% [5]. This small increase of 0.07% in the adult population HIV prevalence was due to the relatively small absolute number comprising high-risk groups that made up a small fraction of the total population even though these groups have much higher HIV prevalence. As the fraction of high-risk groups relative to the total population is generally estimated to be small in India, we assumed that to estimate HIV prevalence in the adult population the antenatal rate would have to be increased by an absolute value of 0.1–0.2% for under-represented high-risk groups in Andhra Pradesh, Karnataka and Tamil Nadu, and 0.15-0.3% in Maharashtra due to the possibility of a higher proportion of high-risk groups in this state. To guard against underestimation, we used higher estimates for the absolute contribution of under-represented high-risk groups to the population HIV prevalence than the 0.07% that we found in the Guntur study.

Using the adjusted sentinel surveillance antenatal HIV prevalence based on the above assuptions for HIV positive referrals and SLI distribution, and under-represented high-risk groups among antenatal women, we calculated the low and high plausible estimates of HIV burden in Andhra Pradesh, Karnataka, Maharashtra and Tamil Nadu.

Although two previously published studies from Tamil Nadu [9,10] and one unpublished study from Karnataka [11] have attempted comparison of population-based and antenatal HIV prevalence, these studies did not specifically analyse data to compare HIV burden estimates with the complete sentinel surveillance method versus the population-based method, nor did these studies analyse data in detail to understand the potential biases in the antenatal HIV estimates as was done in the Guntur study [5]. In addition, due to serious limitations in these studies related to inadequate power because of small sample size, bias in sampling methodology, and poor participation rate, reliable conclusions about the antenatal versus population-based comparison are difficult [5]. Therefore, data from these studies could not be used for the extrapolations that we attempted.

As the coverage of prevention of mother to child transmission (PMTCT) HIV services is increasing in the south Indian states, we also studied the PMTCT data from Andhra Pradesh to assess if these data could be a viable substitute for the annual antenatal sentinel surveillance cycle.

## Results and discussion

### HIV estimates from STI clinics

The original assumption behind including HIV estimates from STI clinics in the sentinel surveillance method of HIV estimation in India was that this would cover the hidden high-risk groups, particularly among men, not reflected in the HIV prevalence in antenatal women or know high-risk groups [2,7]. Interestingly, this approach has not been used elsewhere in the world for estimating HIV burden. Comparisons in the Guntur study revealed that the antenatal HIV estimate from public hospital sentinel surveillance itself exceeded the population-based HIV estimate adjusted for the under-represented high-risk groups even [5]. Addition of the HIV estimate from STI clinic caused a further major overestimation. Assuming that 6% of the adult population gets STI every year and applying the high HIV prevalence among STI patients from large public sector sentinel hospitals, as is done in India currently [7], adds a huge extra HIV component to the estimate. For example, in Andhra Pradesh the 22.8% median HIV prevalence in the STI sentinel sites in 2005 resulted in an estimate of 541,066 persons 15–49 years old with HIV, which was 37.3% of the total 1.45 million estimate for the state, the remainder comprising of 895,486 persons from the antenatal sentinel estimate and 15,385 persons among know high-risk groups [5]. This big contribution from STI sentinel sites was mainly responsible for the much higher total estimated HIV prevalence of 3.35% among adults in Andhra Pradesh with the sentinel surveillance method as compared with a median HIV prevalence of 2% among antenatal surveillance sites in 2005 [5]. Similarly, with the sentinel surveillance based HIV estimation method used in India, the STI component made up over a third of the total HIV estimate for Karnataka in 2005 (STI HIV prevalence 13.6%, antenatal HIV prevalence 1.25%), about a third for Maharashtra (STI HIV prevalence 10.4%, antenatal HIV prevalence 1.25%), and about half for Tamil Nadu (STI HIV prevalence 9.2%, antenatal HIV prevalence 0.5%) [2]. These observed high HIV prevalence in the STI sentinel surveillance are to a large degree due to the location of sentinel STI clinics mostly at large medical college or district headquarters hospitals that get advanced STI patients by referral [12].

Therefore, we suggest that the STI component should be eliminated from the HIV estimation method used in India for the states with a generalised epidemic, defined as antenatal HIV prevalence of more than 1% [13]. For the states in India where the HIV epidemic is not generalised, an informed assessment is needed whether the currently used extrapolation from the sentinel STI clinics to estimate HIV burden is appropriate or an alternate method may be more suitable.

### Referral of HIV patients to public hospitals

The Guntur study presented evidence from public hospital antenatal clinic attendees that HIV positive and suspect women were being referred by private practitioners to the public hospitals [5]. This was the most likely reason for an HIV prevalence among women who used antenatal care in public hospitals that was over two times that among all women needing antenatal care within the same socioeconomic strata in the Guntur population-based sample [5]. Although the confidence intervals for this comparison were wide due to the relatively small number of women who were pregnant within the last 2 years and HIV positive in this study, a similar trend of substantially higher HIV prevalence among both men and women using public hospitals versus other options for general health services within the same socioeconomic strata was also seen in the population-based sample. The latter had much larger numbers of HIV positive cases among them for comparison, suggesting that the antenatal trend seen was not be an aberration. For those involved with health care in India, the common practice of referral of HIV positive and suspect persons by private practitioners and facilities to public hospitals is a widely acknowledged reality, as the private sector for the most part does not prefer to deal routinely with HIV positive patients, though systematic evidence for this has not been published yet. Another contributing factor to HIV positive persons gravitating to public hospitals in India is that among the minority of private practitioners willing to deal with HIV positive patients the cost of accessing health services from them generally becomes so high that many of these patients end up seeking services at public hospitals.

With the very limited systematic data available from India on this issue, it is difficult to comment confidently at this stage about the magnitude of the upward distortion in HIV prevalence among antenatal women going to public hospitals that is being caused in India generally due to referral of HIV positive and suspect women to public hospitals. The magnitude of this may be lower or higher than the doubling effect on HIV prevalence among antenatal women going to public hospitals as compared with all pregnant women in the same socioeconomic strata that was observed in the Guntur study. In any case, given the widespread practice of referral of HIV positive and suspect persons to public hospitals in India, it seems highly likely that this distortion would not be insignificant. To be cautious, as compared with the Guntur trend, we conservatively assumed for our subsequent extrapolations in this paper that this distortion may be causing an increase of HIV prevalence in public hospital antenatal clinic attendees in the range of 1.25 to 1.5 times the rate that would have been seen without this distortion.

### Socioeconomic profile of public antenatal care users

The antenatal care utilisation patterns from the nationwide Reproductive and Child Health District Level Household Survey of 2002–2004 [8] for the 6 high HIV prevalence states of India (according to NACO) and all 10 of the other states with population more than 25 million in 2005 are summarized in Table 1. Antenatal care was sought by 37.4% to 99.3% of the women in the different states, which included 3.9–29.7% from large public sector hospitals equivalent to those that participate in the sentinel surveillance and data from which are used currently by NACO for HIV estimation in India. An additional 1.9–44.9% women reported seeking antenatal care from other public sector health facilities, this proportion exceeding for several states the proportion using large public sector hospitals. This included 0.2–7.2% women who reported seeking antenatal care from medium size community health centres or rural hospitals, a category that is included in sentinel surveillance in some states but data from these are not yet used for estimating HIV in India. Antenatal care was sought from any public sector facility by 5.8–60.7% of the women in the different states. In the three south Indian states of Andhra Pradesh, Karnataka and Tamil Nadu, which together accounted for 48% of the total HIV burden estimated in India with the sentinel surveillance method in 2005, women belonging to the lower half of a standard of living index (SLI) were over-represented (61–70.4%) among those who sought antenatal care from the large public hospitals data from which are used for estimating HIV burden in India (Table 1). The other south Indian state of Kerala also had a similar over-representation of lower SLI among women seeking antenatal care at large public hospitals. In the west Indian state of Maharashtra, the other state contributing substantially to the HIV burden in India (24%), only 14.8% women reported seeking antenatal care at large public hospitals, and lower SLI was not over-represented among them. The two small north-eastern high-prevalence states of Manipur and Nagaland, contributing 1% to the HIV estimate in India, had under-representation of lower SLI among women seeking antenatal care in large public hospitals. Representation of lower SLI was generally higher for women seeking antenatal care from any public sector facility versus those seeking from large public hospitals.

In the population-based sample in the Guntur study we observed that the HIV prevalence was 2.3 times in women belonging to the lower half of SLI as compared with those belonging to the upper half [5]. Because data on this relationship are not available from other parts of India, for our subsequent extrapolations in this paper we conservatively assumed that the Guntur estimate may be the upper limit and that this ratio may range between 1.5 and 2.3.

### Extrapolations to four major states

Using conservative assumptions, which seem reasonably plausible, to adjust the 2005 sentinel surveillance antenatal HIV prevalence in Andhra Pradesh, Maharashtra, Karnataka and Tamil Nadu, the indicative estimates of the HIV burden in the 15–49 years age group are shown in Table 2. Our cautious extrapolations revealed that as compared with the NACO sentinel surveillance based estimate of 1.45 million persons 15–49 years old with HIV in Andhra Pradesh, our estimate of the plausible range was 0.54 to 0.73 million (Table 2). For Andhra Pradesh, Maharashtra, Karnataka and Tamil Nadu combined, our estimate of the plausible range was 1.50 to 2.01 million as compared with the sentinel surveillance based estimate of 3.69 million by NACO (Table 2). Our calculations suggest that the official estimate has a minimum overestimation of 1.68 million for these four states, a figure which makes up 33% of the total 5.15 million persons 15–49 years old estimated by NACO to have HIV in India in 2005 [2]. If we assume that the remaining 1.5 million official estimate for the other Indian states combined is not an over- or underestimate, the actual estimate for India may be 3–3.5 million, with the official estimate of 5.2 million possibly grossly overestimating the HIV burden in India. Clearly, our estimates are only indicative at this stage, though these are based on assumptions that seem plausible as explained earlier. Our assumptions and HIV estimates for the four major Indian states would need to be verified by well-designed population-based studies in other parts of India. In addition, a more informed assessment of the official HIV estimates for the other Indian states, the majority of which do not have generalised epidemics, would also be useful.

It is important to note that if the 2005 antenatal sentinel surveillance HIV prevalence for Andhra Pradesh, Maharashtra, Karnataka and Tamil Nadu were applied directly to the adult populations 15–49 years old in these states, the total HIV burden would be 2.1 million. This is very close to our high plausible estimate of 2 million for these four states. Therefore, the major reduction in our estimate from the 3.7 million estimate by NACO for these four states is due to elimination of the component from STI clinics in our estimates that is included in the method used by NACO. Even our low plausible estimate of 1.5 million for the four states combined is only about a quarter lower than the estimate of 2.1 million that would be obtained by direct application of sentinel surveillance ANC rates, which is quite compatible with the proportional downward revisions that UNAIDS did recently for the HIV burden estimates for several sub-Saharan African countries due to the availability of population-based surveys that showed lower HIV prevalence than antenatal surveillance rates [1].

NACO has recently set-up an expert committee to examine issues related to HIV estimates for India that is expected to give its report in early 2007 [16]. In light of data from the Guntur population-based study [5] and the plausible impact of these data on the HIV estimates for India as reported in this paper, a strategy to critically examine the currently used official method of HIV estimation in India seems indicated that should take into account the best available evidence. Efforts to improve the HIV burden estimates based on population-based data have so far been undertaken mostly for sub-Saharan Africa [1,17-19], but it is time that this be attempted for India too as it is currently estimated to have the highest HIV burden in the world [1]. The latter may not actually be the case if our extrapolations in this paper are verified by further population-based studies. The approaches that we used to understand the population-based versus the sentinel surveillance based estimates of HIV burden in the Guntur study [5], and their extrapolation to major HIV states in this paper, offer pointers to the types of analyses that could be useful for interpreting the HIV data from the National Family Health Survey that is being currently conducted in India.

### Implications for antenatal sentinel surveillance

The proportion of women utilising antenatal care at public hospitals that are included in the sentinel surveillance in India is quite small, ranging between 4–31% for the high HIV prevalence states or those with population more than 25 million (Table 1). In addition, a systematic comparison of the HIV pattern in public hospital antenatal care users versus the population-based pattern observed in the large-sample rigorous methodology study in Guntur revealed that although the HIV prevalence in all pregnant women and the adult population were similar, there were significant biases in the current public hospital based sentinel surveillance [5]. This implies that the annual antenatal HIV surveillance as done in India currently has major limitations regarding generalisability of the findings to either adult women or all adults, which raises two related questions. First, is it possible to improve the generalisability of the antenatal sentinel surveillance data? Second, are there alternatives to the annual sentinel surveillance cycle that might yield more reliable data? These are addressed in the following paragraphs.

Achieving a representative sample of all pregnant women in the sentinel surveillance in India would require inclusion of the full range of private sector antenatal care and also women who do not get antenatal care (Table 1). This may not be feasible given the complexity and variety of private health care in India. However, a slight improvement would be feasible if antenatal clinics in the public sector other than the large hospitals are also included in the sentinel surveillance based HIV estimation. Antenatal sentinel surveillance data from medium size hospitals in the public sector are collected in some states in India currently but are not used for HIV burden estimation. This can be easily done and should be considered. Still, however, direct generalisation of HIV data from the public hospital antenatal sentinel surveillance will pose major problems due to the biases described earlier. Extrapolation of these data to the adult population will require use of correction factors from periodic strategically planned population-based studies, using approaches such as the one attempted in the Guntur study [5].

As the sample size of 400 in each district for the annual antenatal sentinel surveillance cycle is quite small, the larger number of antenatal women receiving PMTCT services at public hospitals may offer a more reliable alternative for tracking HIV in states where this coverage is good and the participation rates are high. For example, over 210,000 women received HIV testing as part of PMTCT services in Andhra Pradesh in the latest 2005–06 annual cycle at 37 antenatal clinics at medical colleges and district headquarters hospitals, covering 89% of the new antenatal registrations at these clinics (Table 3). The much larger sample sizes for the PMTCT services enable tighter confidence intervals for HIV prevalence than are possible with the small sample annual sentinel surveillance, thereby enabling a more reliable estimate of HIV prevalence if accompanied with a high participation rate (Table 3). This becomes particularly important for district-level data and planning, which is receiving increasing attention in the context of the anticipated decentralised planning in the upcoming next phase of India' National AIDS Control Programme [20]. The importance of having tighter confidence intervals for more reliable interpretation of antenatal HIV data has been emphasised recently [19,21]. The other major HIV states, Karnataka, Maharashtra and Tamil Nadu, also have large numbers of women receiving HIV testing as part of PMTCT services, ranging from 110,000 to 330,000 in the latest year, with 80%, 85% and 93% participation rates among new antenatal registrations at participating clinics (data from respective AIDS Control Societies). PMTCT coverage is increasing further in several states. It is important to note though that for the PMTCT HIV data to be useful, participation rates in the range of 85–90% at antenatal clinics would be needed, as lower participation rates would pose problems in interpretation due to the possibility of selection bias. The large PMTCT sample size if accompanied with adequate participation rate could outweigh the benefit of a consecutive small sample as attempted in the annual sentinel surveillance cycle. The biases that make women attending public hospital antenatal clinics unrepresentative of adult women or all adults would remain with the PMTCT sample from public hospitals, but this would be no worse than for the smaller annual sentinel surveillance sample from these same hospitals. Therefore, India should consider further strengthening the PMTCT data for tracking antenatal HIV prevalence in the states where this coverage is good, and possibly abort the relatively small sample annual antenatal surveillance cycle in these states, as the former can potentially offer more reliable data. For population HIV estimates, the correction factors derived from periodic population-based studies, as mentioned above, would need to be applied to the PMTCT antenatal HIV data in these states.

### End note

When conventional beliefs are questioned this often meets resistance initially, as happened recently with findings from the Women's Health Initiative research study in the United States, which provoked strong reactions in the scientific community and the public, including "disbelief, disagreement, discouragement, and a fair measure of dissention and disharmony" [22]. Although our study does not have as big a scope as this cited example, there may be resistance in some circles to give due consideration to our finding that the current official estimate of HIV burden in India is likely a gross overestimate. However, it would be prudent for scientists and policy makers to consider this possibility and examine it objectively, as disregarding this simply because it may pose inconvenient issues related to revising official estimates and implications for planning would not be in the best interest of society at large or HIV control in India.

## Conclusion

• A large-sample methodologically rigorous study in a south Indian district revealed that the population-based estimate of HIV burden even after adjusting for under-represented high-risk groups was 2–3 times lower than the estimate based on the official sentinel surveillance based method used in India, and systematically documented the reasons for this difference.

• Plausible and cautious extrapolation of these findings to four major states, which are estimated to contribute the bulk of HIV burden in India, suggested that the HIV burden among 15–49 years old persons in these states may be in the range of 1.5–2 million instead of the 3.7 million estimated by the official sentinel surveillance based method, which would drop the total 5.2 million estimate for India to 3–3.5 million.

• The biggest upward distorter in the official sentinel surveillance method of estimating HIV burden is the inclusion of highly biased HIV data from public hospital STI clinics, which should be dropped from this method in the Indian states that have a generalised HIV epidemic.

• Better understanding of the magnitude of biases in public hospital based antenatal sentinel surveillance, including referral of HIV positive/suspect persons by the private to the public sector and the socioeconomic profile of women using public hospitals, and their effect on the HIV burden estimation in different parts of India is needed.

• Periodic, strategically planned, and scientifically sound population-based studies of HIV will be needed in India to get an accurate estimate of HIV burden and its evolving trend for informed planning of HIV control in India.

## Competing interests

The author(s) declare that they have no competing interests.

## Authors' contributions

LD conceived this report, led the analysis, and drafted the manuscript. VL contributed to the interpretation. GAK contributed to the statistical analysis and interpretation. RD contributed to the analysis and interpretation. All authors approved the final version of the manuscript.

## Pre-publication history

The pre-publication history for this paper can be accessed here:



## Figures and Tables

**Table 1:** Summary of public sector antenatal care utilization in Indian states from the nationwide Reproductive and Child Health Household Level Survey of 2002–2004.

							SLI of women who used government/municipal hospital for antenatal care		SLI of women who used any public sector facility for antenatal care			
									
**States**	2005 population* (millions)	2005 population 15–49 years* (millions)	Sample of women who provided data regarding antenatal care	% who received antenatal care	Percent who received antenatal care at public sector government/municipal hospital†	Percent who received antenatal care at other public sector facilities‡	% in lower SLI half§	% in upper SLI half§	% in lower SLI half§	% in upper SLI half§	2005 median antenatal sentinel surveillance HIV prevalence¶(%)	2005 HIV estimate15–49 years¶(1000s)
**High prevalence states (I)**#												
Andhra Pradesh	80.0	42.7	5,476	95.1	23.9	9.0	62.4	37.6	64.1	35.9	2.00	1,452
Karnataka	56.0	30.0	7,543	90.5	24.7	13.4	61.0	39.0	65.3	34.7	1.25	640
Maharashtra	104.2	55.0	9,284	94.0	14.8	29.5	43.5	56.5	63.0	37.0	1.25	1,232
Tamil Nadu	65.2	36.3	7,480	99.3	29.7	26.2	70.4	29.6	74.5	25.5	0.50	366
High prevalence states (II)**												
Manipur	2.3	1.2	3,592	67.1	28.5	23.1	34.1	65.9	42.7	57.3	1.25	32
Nagaland	2.1	1.1	2,326	56.4	21.6	13.3	38.2	61.8	48.1	51.9	1.63	26
**Other south Indian state**††												
Kerala	33.0	18.3	3,355	99.7	30.6	2.6	68.1	31.9	67.8	32.2	0.25	68
**Other Indian states (I)**‡‡												
Gujarat	53.8	29.0	7,349	88.4	7.2	19.0	52.1	47.9	65.8	34.2	0.25	103
Punjab	26.0	13.8	4,552	90.9	25.7	22.4	56.2	43.8	63.7	36.3	0.13	28
West Bengal	85.0	45.2	5,024	90.4	15.8	44.9	44.7	55.3	70.0	30.0	0.84	395
**Other Indian states (II)**§§												
Madhya Pradesh	65.2	31.6	15,909	75.1	14.3	15.5	36.7	63.3	50.0	50.0	0.25	99
Orissa	39.1	20.3	9,178	76.4	23.9	28.4	40.9	59.1	54.4	45.6	0.25	84
Rajasthan	61.8	29.2	13,505	67.1	13.9	24.5	36.4	63.6	49.0	51.0	0.13	87
**Other Indian states (III)**¶¶												
Bihar	90.7	41.2	18,304	37.4	3.9	1.9	54.1	45.9	54.7	45.3	0##	87
Jharkhand	29.5	14.2	7,437	51.2	7.7	6.2	51.4	48.6	53.5	46.5	0.13	33
Uttar Pradesh	181.7	83.6	30,986	55.8	12.0	14.0	38.8	61.2	52.8	47.2	0##	171

*The states shown in this table are estimated to have 89% of India's population; population in 2005 estimated by calculating the exponential annual growth rate for each state from the 1991 and 2001 censuses [14,15], and using 90% of this annual estimate for growth from 2001 to 2005, except for Tamil Nadu and Kerala for which the estimate from the last decade was used as the growth rate was already very low between the two censuses.

†Considered equivalent to large public sector hospitals, the category from which sentinel surveillance data are used for estimating HIV burden in each state.

‡All other categories of public sector health facilities, including primary health centres and sub-centres, which are not included in the sentinel surveillance based HIV estimation.

§SLI is standard of living index based on living conditions and assets, which was similar to but not exactly the same as the SLI used in our population-based study; cut-off values to define lower and upper halves calculated based on the distribution of SLI in all members of the sampled households in each state, as the sampling used in this survey was expected to yield a sample representative of the population of each state [8].

¶Median HIV prevalence and HIV estimate in adults for 2005 as calculated by NACO [2].

#High prevalence states according to NACO, which have large population; Andhra Pradesh, Karnataka and Tamil Nadu are in south India and Maharashtra is in west India.

**High prevalence states according to NACO, which have small population; these two states are in north-east India.

††Kerala, the other south Indian state shown separately, as it has almost complete antenatal coverage and the SLI distribution of women using public sector antenatal care is similar to the other south Indian states but is not considered to be a high HIV prevalence state.

‡‡Other Indian states with a high total level of antenatal care coverage.

§§Other Indian states with a medium total level of antenatal care coverage.

¶¶Other Indian states with a relatively low total level of antenatal care coverage.

##If the median HIV prevalence for a state is zero at sentinel surveillance antenatal clinics, in the NACO method of calculating HIV burden this is replaced with the average rate for that state during the last three years, but if this average rate is also zero then the average rate for the low prevalence states for 2003 is used [7].

**Table 2:** Estimation of HIV burden in four major Indian states based on correction factors derived from population-based study.

				**Plausible estimate (low)**					**Plausible estimate (high)**				
				
				**Change needed in antenatal HIV prevalence to estimate population prevalence **(% of antenatal HIV prevalence)					**Change needed in antenatal HIV prevalence to estimate population prevalence **(% of antenatal HIV prevalence)				
									
**State**	2005 population 15–49 years* (millions)	**2005 median antenatal sentinel surveillance HIV prevalence**† (%)	**2005 HIV estimate 15–49 years**† (millions)	To adjust for HIV referrals to public sector hospitals‡	To adjust for the socio-economic profile of public sector hospital users§	To adjust for under-represented high-risk groups¶	**Composite correction factor for antenatal HIV prevalence to estimate population prevalence**	**2005 HIV estimate 15–49 years**# (millions)	To adjust for HIV referrals to public sector hospitals‡	To adjust for the socio-economic profile of public sector hospital users§	To adjust for under-represented high-risk groups¶	**Composite correction factor for antenatal HIV prevalence to estimate population prevalence**	**2005 HIV estimate 15–49 years**# (millions)
Andhra Pradesh	42.7	2.00	1.45	-33	-9	+5	0.63	0.54	-20	-5	+10	0.85	0.73
Karnataka	30.0	1.25	0.64	-33	-8	+8	0.67	0.25	-20	-4	+16	0.92	0.35
Maharashtra	55.0	1.25	1.23	-33	+5	+12	0.84	0.58	-20	+3	+24	1.07	0.73
Tamil Nadu	36.3	0.50	0.37	-33	-14	+20	0.73	0.13	-20	-8	+40	1.12	0.20
**Total**			**3.69**					**1.50**					**2.01**

*Population in 2005 estimated by calculating the exponential annual growth rate for each state from the 1991 and 2001 censuses [14,15], and using 90% of this annual estimate for growth from 2001 to 2005, except for Tamil Nadu for which the estimate from the last decade was used as the growth rate was already very low between the two censuses.

†Median HIV prevalence and HIV estimate in adults for 2005 as calculated by NACO [2].

‡In Guntur district the rate in both the lower and upper SLI halves was over 2 times in women who utilised antenatal care at public sector hospitals than the average for each SLI half [5], which can be related to the common practice of referral of HIV positive or suspect persons from the private to the public health system; as this referral pattern is common we assumed it to be broadly similar in the four states but of a lower magnitude than observed in Guntur district; we used a conservative assumption of a 1.5 times increase in HIV prevalence in women utilising care at public sector hospitals due to this referral for our low estimate that would require a 33% downward adjustment of the antenatal HIV prevalence, and used a still more conservative assumption of only a 1.25 times increase in the antenatal HIV prevalence due to referrals for our high estimate that would require a 20% downward adjustment of this HIV prevalence.

§Based on the SLI distribution of women using public sector hospitals for antenatal care in each state (Table 1); if representation of the lower half of SLI was higher for a state, a downward adjustment was calculated for the antenatal HIV prevalence based on what the rate would have been if the two SLI halves were equally represented, and vice versa; in our population- based sample the ratio of HIV prevalence among women in the lower and upper halves of SLI was 2.3, and we used this assumption for our low estimate; we used a more conservative ratio of 1.5 for our high estimate.

¶Inclusion of under-represented high-risk groups increased our population-based HIV prevalence in Guntur district from 1.72% to 1.79% [5], a small increase of 0.07%; we assumed a population HIV prevalence of 0.1% due to under-represented high-risk groups for our low estimate and 0.2% for our high estimate for Andhra Pradesh, Karnataka and Tamil Nadu; for Maharashtra, due to the possibility of a higher proportion of high-risk groups, we assumed 0.15% and 0.3% rates respectively due to their under-representation; the upward adjustment needed in the antenatal HIV prevalence to accommodate this was calculated by dividing these assumed rates with the median antenatal rate for each state.

#Calculated by multiplying the antenatal HIV prevalence with the composite correction factor and applying this to the total population 15–49 years old.

**Table 3:** HIV testing in antenatal women as part of PMTCT services in the public health system versus annual sentinel surveillance in Andhra Pradesh.

	Antenatal clinic outpatient* April 2005 – March 2006					**Sentinel surveillance in antenatal clinic† August – October 2005**		
	
**Districts in three regions of Andhra Pradesh (number of antenatal clinics providing PMTCT services)**	**Number of new antenatal registrations**	**Number who had HIV test as part of PMTCT services**	**% of new registrants who had HIV testing as part of PMTCT services**	**Number HIV positive**	**% HIV positive (95% confidence interval)**	**Number of participants**	**Number HIV positive**	**% HIV positive (95% confidence interval)**
**Coastal region (14)**	**87,855**	**76,434**	**87.0**	**1,812**	**2.39 ** **(2.28–2.50)**‡	**3,600**	**85**	**2.47 ** **(1.96–2.98)**‡
East Godavari (2)	15,631	12,751	81.6	448	3.51 (3.19–3.83)	400	12	3.00 (1.33–4.67)
Guntur (2)	14,494	14,166	97.7	376	2.65 (2.39–2.92)	400	12	3.00 (1.33–4.67)
Krishna (2)	12,045	11,452	95.1	377	3.29 (2.96–3.62)	400	8	2.00 (0.63–3.37)
Nellore (1)	5,373	4,863	90.5	87	1.79 (1.42–2.16)	400	6	1.50 (0.31–2.69)
Prakasam (1)	2,797	2,747	98.2	65	2.37 (1.80–2.94)	400	10	2.50 (0.97–4.03)
Srikakulam (1)	2,529	2,458	97.2	46	1.87 (1.33–2.41)	400	6	1.50 (0.31–2.69)
Visakhapatnam (3)	22,358	17,358	77.6	232	1.34 (1.17–1.51)	400	10	2.50 (0.97–4.03)
Vizianagaram (1)	6,603	4,910	74.4	44	0.90 (0.64–1.16)	400	8	2.00 (0.63–3.37)
West Godavari (1)	6,025	5,729	95.1	137	2.39 (1.99–2.79)	400	13	3.25 (1.51–4.99)
**Rayalaseema region (7)**	**36,334**	**33,523**	**92.3**	**342**	**0.99 ** **(0.88–1.10)**‡	**1,600**	**21**	**1.35 ** **(0.78–1.92)**‡
Anantapur (2)	6,424	6,382	99.3	57	0.89 (0.66–1.12)	400	7	1.75 (0.46–3.04)
Chittoor (2)	14,791	12,863	87.0	135	1.05 (0.87–1.23)	400	5	1.25 (0.16–2.34)
Cuddapah (1)	3,618	3,268	90.3	29	0.89 (0.57–1.21)	400	3	0.75 (0.00–1.60)
Kurnool (2)	11,501	11,010	95.7	121	1.10 (0.91–1.29)	400	6	1.50 (0.31–2.69)
**Telangana region (16)**	**113,621**	**101,174**	**89.0**	**1,200**	**1.64 ** **(1.56–1.72)**‡	**4,000**	**78**	**1.95 ** **(1.52–2.38)**‡
Adilabad (1)	2,420	2,256	93.2	17	0.75 (0.39–1.11)	400	7	1.75 (0.46–3.04)
Hyderabad (5)	62,586	53,467	85.4	531	0.99 (0.91–1.07)	400	8	2.00 (0.63–3.37)
Karimnagar (1)	2,509	2,393	95.4	89	3.72 (2.96–4.48)	400	9	2.25 (0.80–3.70)
Khammam (1)	5,232	4,495	85.9	110	2.45 (2.00–2.90)	400	14	3.50 (1.70–5.30)
Mahabubnagar (1)	2,778	2,736	98.5	21	0.77 (0.44–1.10)	400	1	0.25 (0.00–0.74)
Medak (1)	3,995	3,755	94.0	48	1.28 (0.92–1.64)	400	8	2.00 (0.63–3.37)
Nalgonda (1)	1,980	1,906	96.3	63	3.31 (2.51–4.11)	400	11	2.75 (1.15–4.35)
Nizamabad (1)	4,468	4,447	99.5	57	1.28 (0.95–1.61)	400	3	0.75 (0.00–1.60)
Rangareddy (1)	3,138	2,744	87.4	19	0.69 (0.38–1.00)	400	7	1.75 (0.46–3.04)
Warangal (3)	24,515	22,975	93.7	245	1.07 (0.94–1.20)	400	10	2.50 (0.97–4.03)
**Total**	**237,810**	**211,131**	**88.8**	**3,354**	**1.85 ** **(1.79–1.91)**‡	**9,200**	**184**	**2.07 ** **(1.78–2.36)**‡

*These data are from the 37 antenatal clinics at medical colleges and district headquarter hospitals that started PMTCT services in 2002, and do not include PMTCT services that were started in late 2005 at 64 additional antenatal clinics in smaller locations.

†These data are from the 23 medical college and district headquarter hospital antenatal clinics, one in each district, that are used by NACO for calculating HIV estimates, and do not include data from 21 antenatal clinics at smaller locations; additional data from the Hyderabad medical college antenatal clinic on a sample of 15–24 years old women only not included; these 23 clinics are a subset of the 37 clinics for which PMTCT data are shown.

‡These composite HIV prevalence are weighted by the population of 15–49 years old women in each district [14].
